# Localized and triggered release of oxaliplatin for the treatment of colorectal liver metastasis

**DOI:** 10.7150/jca.48528

**Published:** 2020-10-12

**Authors:** Venkateswara R Gogineni, Dilip R Maddirela, Wooram Park, Jaidip M Jagtap, Abdul K Parchur, Gayatri Sharma, El-Sayed Ibrahim, Amit Joshi, Andrew C Larson, Dong-Hyun Kim, Sarah B White

**Affiliations:** 1 Departments of Radiology & *Biomedical Engineering, Medical College of Wisconsin, Milwaukee, WI.; 2 Department of Radiology, Northwestern University, Chicago, IL.

**Keywords:** hybrid liposome-magnetic nanoparticles, oxaliplatin, colorectal liver metastases

## Abstract

**Purpose:** The aim of this study was to develop and evaluate a liposome formulation that deliver oxaliplatin under magnetic field stimulus in high concentration to alleviate the off-target effects in a rat model of colorectal liver metastases (CRLM).

**Materials and Methods:** Hybrid liposome-magnetic nanoparticles loaded with Cy5.5 dye and oxaliplatin (L-NIR- Fe_3_O_4_/OX) were synthesized by using thermal decomposition method. CRLM (CC-531) cell viability was assessed and rats orthotopically implanted with CC-531 cells were treated with L-NIR-Fe_3_O_4_/OX or by drug alone via different routes, up to 3 cycles of alternating magnetic field (AMF). Optical and MR imaging was performed to assess the targeted delivery. Biodistribution and histology was performed to determine the distribution of oxaliplatin.

**Results:** L-NIR-Fe_3_O_4_/OX presented a significant increase of oxaliplatin release (~18%) and lower cell viability after AMF exposure (*p*<0.001). Optical imaging showed a significant release of oxaliplatin among mesenteric vein injected (MV) group of animals. MR imaging on MV injected animals showed R2* changes in the tumor regions at the same regions immediately after infusion compared to the surrounding liver (*p*<0.001). Biodistribution analysis showed significantly higher levels of oxaliplatin in liver tissues compared to lungs (*p*<0.001) and intestines (*p*<0.001) in the MV animals that received AMF after L-NIR- Fe_3_O_4_/OX administration. Large tumor necrotic zones and significant improvement in the survival rates were noted in the MV animals treated with AMF.

**Conclusion:** AMF triggers site selective delivery of oxaliplatin at high concentrations and improves survival outcomes in colorectal liver metastasis tumor bearing rats.

## Introduction

The American Cancer Society estimates that in the United States, colorectal cancer is the third leading cause of cancer-related deaths in men and women alone and second most common when combined and expecting 51,020 deaths during 2019 1. Surgical removal of primary tumor offers survival benefit beyond 5 years in 50% of the patients 2, 3. Further, only 25% of patients are suitable for surgical resection because of the synchronous metastatic disease, inaccessible locations and small size of the lesions 4. According to the National Comprehensive Cancer Network (NCCN) guidelines, the first line therapy for treatment of metastatic colorectal cancer is systemic chemotherapy 5. These regimens are difficult to tolerate, resulting in only 63% of patients receiving a full 12 cycles 6. Recent advances in minimally invasive locoregional treatments have gained significance for the non-surgical management and curative treatment of small HCC lesions. The initial results from studies evaluating drug eluting beads using chemotherapy have shown great promise 7, 8, but their use has not been widely adopted. The tolerability of DEBIRI was poor, with 19% having severe side effects 9. Because the beads require the drug to bind via ionic bonds, once infused, the drug elutes off of the bead very rapidly, resulting in higher systemic drug concentrations and systemic side effects 10. Therefore, different drug combinations, and drug delivery platforms are needed to decrease systemic delivery and toxicities. Transarterial therapies offer an alternative for patients that cannot tolerate systemic therapy and are not resection, or SBRT candidates. Radioembolization can be utilized; however, recent results demonstrated no overall survival benefit from its integration with systemic therapy 11. Therefore, this bears the question whether re-examination of a non-radiation based local therapy would benefit patients.

Oxaliplatin, a third generation platinum based chemotherapy agent prevents cellular replication and leads to cell death 12. Oxaliplatin has a unique molecular structure, making it more efficient than cisplatin at preventing DNA replication per equal number of DNA bound to drug 13. Oxaliplatin alone and in combination with 5FU increase survival in patients with colorectal cancer 14, 15. However, oxaliplatin-induced neurotoxicity results in dose limiting toxicity in 81.5-98% of patients 16. Effective oxaliplatin delivery systems were initiated and nanoparticle platforms are advantageous due to reduced systemic toxicities 17, 18. Many nanoparticle drug formulations including liposomes have been validated clinically and have received FDA approval 19, 20. Liposomes are well suited to the dual-delivery of hydrophobic and hydrophilic therapeutics, which make liposomes excellent therapeutic carriers. At present, there are more than 11 liposomal formulations approved for clinical use, with many more in clinical and preclinical development. Iron oxide (Fe_3_O_4_) nanoparticles demonstrate large magnetization and high relaxivity, making them ideal and successful for hyperthermia applications in cancer treatments 21. Combining a liposome with Fe_3_O_4_ allows for envelopment of a drug by the lipid shell.

Therefore, the objective of this study was to develop and evaluate an Fe_3_O_4_ based liposome formulation to deliver oxaliplatin under heat stimulus driven by an alternating magnetic field (AMF) to alleviate the off-target effects in a rat model of colorectal liver metastases (CRLM). Our approach integrated nanomedicine, molecular MRI, optical imaging, site selective delivery and controlled release to optimize treatment efficacy.

## Materials and Methods

Hybrid liposome-magnetic nanoparticles loaded with Cy5.5 dye and oxaliplatin (L-NIR- Fe_3_O_4_/OX).

### Formulation

Liposome-magnetic nanoparticles were fabricated as uniform Fe_3_O_4_ nanocubes using the thermal decomposition method and transmission electron microscopy (TEM) for evaluation and size determination as described elsewhere 22. The nanocubes and oxaliplatin were encapsulated in sterically stabilized polyethylene glycol (PEG)-coated liposomes forming magnetic liposomes. The loading efficiency and size distribution of the L-NIR-Fe_3_O_4_/OX nanoparticles were determined. The loading efficiency was analyzed using high-performance liquid chromatography (HPLC) according to the previous report 23. The particle size of the liposomes were determined with a Zetasizer Nano-ZS particle analyzer (Malvern Instruments Ltd., Malvern, UK). After exposing nanoparticles to AMF, drug release was quantified at different time points using HPLC.

### Cell Culture and Cytotoxicity Assay

CC-531 cell line was cultured as described elsewhere 24. Cells (3×10^4^) plated in 96 wells were divided into different groups and exposed to an alternating magnetic field (~1.85 kW, 200 kHz) for 5 minutes at every hour for 6 hrs and at 24, 48, 72 and 96 hrs. AMF settings and Fe_3_O_4_ concentration in the liposomes were kept constant at varying oxaliplatin concentrations loaded in the liposomes. For the first 24 hrs, 5 µL of supernatant was collected from the group at 0.25, 0.5, 1, 2 and 24 hrs after administration of the drug/nanoparticle and after each exposure to the AMF. The drug concentration was measured in supernatant using spectrophotometry. Cell viability was examined in each group using trypan blue dye. The percent of cells attached after 24 hrs was determined from counts of the cells removed from the plates by trypsinization as described earlier 25.

### Animal Model

The animal experiments were performed in accordance with the Institutional Animal Care and Use Committee, Medical College of Wisconsin guidelines. Orthotopic tumors were implanted in rats weighing ~400 gm as described earlier 24 and all the rats implanted with cells developed tumors. After one week of implantation, tumor bearing rats (~4-5 mm^3^ tumor volume) were divided randomly into four experimental groups as control/sham group, mesenteric vein (MV) infusion group, MV + AMF trigger group and tail vein (IV) group.

### Optical imaging

Optical imaging in near-infrared (NIR) wavelength range is a non-invasive imaging that can sensitively track biochemical events using fluorescent dyes and nanomaterials. Cy5.5 dye loaded L-NIR-Fe_3_O_4_/OX phantoms or anesthetized rats were imaged by near-infrared (NIR) fluorescence imaging system (λ_ex_/λ_em_=663nm/longpass 700nm) at 100 ms exposure time using the method described elsewhere 26. A 663 nm diode laser (90 mW) with deep cooled intensified charge-coupled device (ICCD) camera (PiMAX, Princeton Instruments, Trenton, NJ) was used for fluorescence imaging. Imaging was performed before and after triggering with the external magnetic field.

### MR Imaging (MRI)

MRI studies were performed using a 9.4T Clinscan MRI scanner (Bruker Biospin, Ettlingen, Germany) with a commercial rat coil (Bruker Biospin, Ettlingen, Germany) and respiratory gating system permitting free-breathing acquisition during MRI measurements (SAII, Stony Brook, NY). MR imaging was performed to monitor the tumor growth and T2* relaxivity was used to quantify the liposomes in the tumor/liver regions. To characterize T2* relaxivity of the liposomes, phantoms were made in four tubes filled with L-NIR- Fe_3_O_4_/OX, with iron-oxide ranging from 0 to 40 ug/ml, in 1% agarose gels. R2* relaxometry was assessed using a gradient-echo T2* mapping sequence. The imaging parameters were: repetition time (TR) = 800 ms, 9 echo times (TE) ranging from 4 ms to 48 ms in 5.5 ms increments, flip angle = 50⁰, matrix = 128×128, field of view = 45×45 mm^2^, slice thickness = 1 mm, acquisition bandwidth = 586Hz/pixel, and #averages = 2, scan time ~1 minute/slice. Signal intensities were fitted to a mono-exponential decaying curve to derive T2* value, from which R2* was measured as the reciprocal of T2*. Least-square fitting was conducted between mean R2* and Fe_3_O_4_ concentration in the imaged phantoms. MRI was performed on animals before and immediately after liposomes infusion, as well as after AMF triggering using T2* imaging protocol as implemented in phantom scan. Stacks of parallel axial and coronal slices covering the liver were acquired using respiratory gated acquisition to avoid breathing artifacts. T2* maps were created for all acquired slices, from which T2* values were measured in the tumors and normal tissues in all slices at three time points: before L-NIR-Fe_3_O_4_/OX infusion, after infusion, and after AMF triggering.

### L-NIR-Fe_3_O_4_/OX infusion

Catheterization of the MV was performed following the general method described earlier 27. In short, the animal was anesthesized to a surgical plane. A median laparotomy was performed and the bowel was externalized. A 2.0 silk tie was placed proximal and distal to the access site. Then, using a 25 G angiocather (Terumo, Somerset, NJ), access was obtained into a distal mesenteric vein. Then, a 0.014” Fathom microwire (Boston Scientific, Marlborough, MA) was advanced through the angiocath and advanced into the portal vein (PV) under fluoroscopic guidance (OEC9800 Plus, GE Healthcare, Salt Lake City, UT). The angiocath was then exchanged for a 22G 8 cm PowerGlide ST^TM^ (BD Bard, Frankling Lakes, NJ), which was advanced into the PV. Digital subtraction angiography was performed confirming that the catheter was in the PV. Each animal was given with 0.5 mL of L-NIR-Fe_3_O_4_/OX (6mg/kg of oxaliplatin) followed by 0.2 mL of saline. The catheter was then removed and the silk suture was used to ligate proximal and distal to the access site. The incision was closed with a two layer closure. An intravenous group of animals (IV) were administered 6 mg/kg oxaliplatin dissolved in saline via bolus tail vein injection.

### AMF induction in rats

Compact induction heating system (EASYHEAT Ambrell Corporation, Scottsville, NY) was used to generate AMF as described elsewhere^28^ in the present study. After liposome infusion, each of the anesthetized rat was inserted into magnetic induction coil in such a way that the abdomen region reside in the coil. AMF triggering was performed for 30 minutes with 470.4 A and 196 KHz maintaining anesthetic depth similar to infusion. The core temperature was monitored during the treatment and animal was allowed to breathe freely. In a separate group of animals (n=5), AMF triggering was performed three times after L-NIR-Fe_3_O_4_/OX infusion with 30 minutes gap between each trigger.

### Histology, tumor necrosis & staining

The animals were euthanized at different time points, and tumors were collected for histologic assessment. Tissue sections were prepared and stained with H&E. Prussian blue staining was performed by the histology core lab to identify regions of Fe_3_O_4_ deposition within the tumor (Sigma-Aldrich, St Louis, MO) 29. Tumor necrosis in H&E sections was determined by recording the longest and transverse dimensions of each tumor and necrosis area as described earlier 29, and these dimensions allowed comparison of treatment efficacy. Nanoparticle distribution in the tissues was visualized by randomly chosen high powered fields within each tissue.

### Biodistribution

Animals were euthanized at different points after saline or L-NIR- Fe_3_O_4_/OX infusion via MV to confirm the localized release of oxaliplatin. Snap frozen tissues collected from animals (lungs, liver and gut) were subjected to acid-digestion, using HNO_3_ following the previously published method 27. The digested tissues were appropriately diluted and sent for ICP-MS analysis of platinum in each tissue.

### Statistical Analysis

R2 and R2* for each phantom model was compared to ICP-MS measured Fe concentration using Spearman's correlation and nonparametric Mann-Whitney U test to compare the pre and post AMF triggering. Student's t-test was performed to compare changes in viability of cells, distributed Fe amounts in organs and differences in percent survivals. A one-way ANOVA was performed for the area of necrosis with post hoc analysis.

## Results

### Synthesis of L-NIR- Fe_3_O_4_/OX

**Figure 1:**
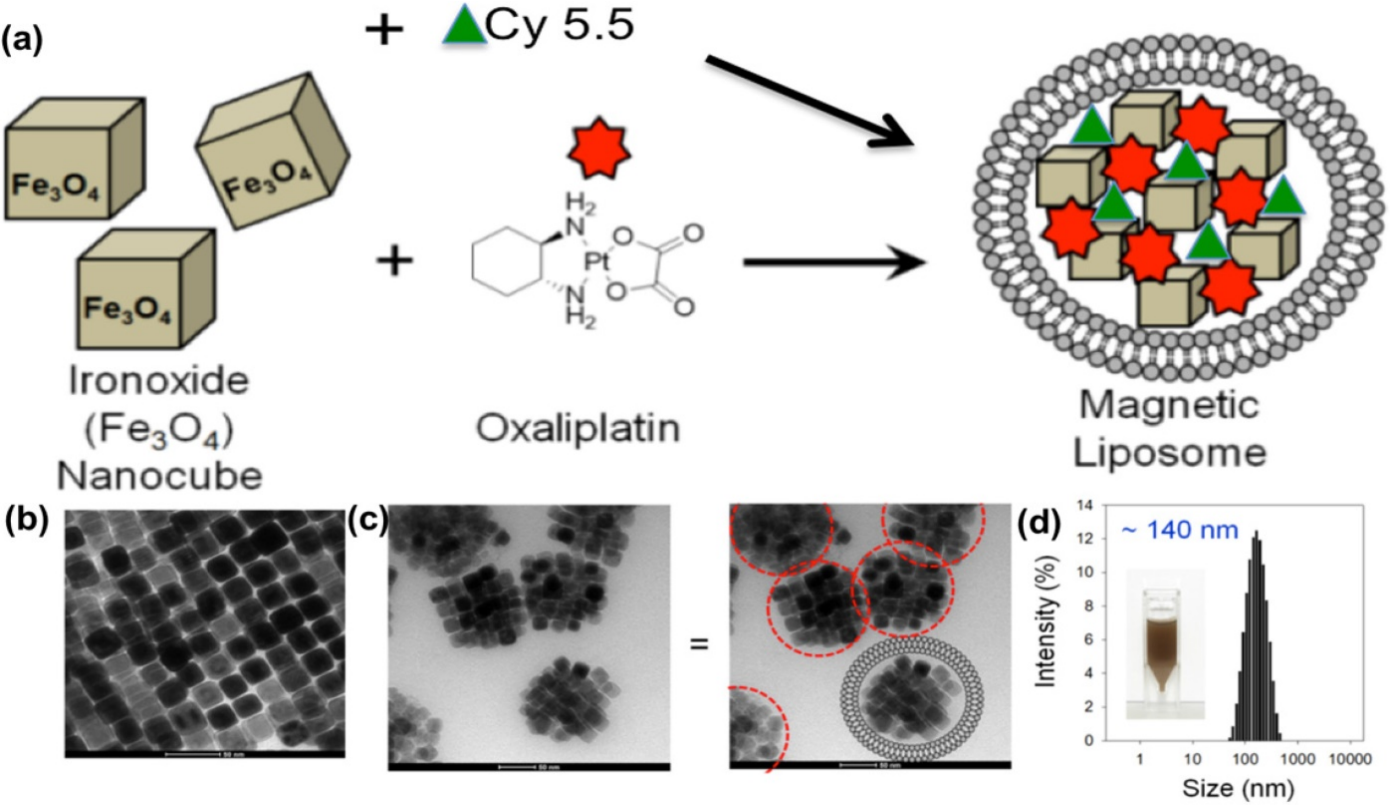
(a) Schematic illustration of oxaliplatin, Cy5.5 and ironoxide nanocubes loaded liposome, (b and c) TEM image of (b) ironoxide nanocubes and (c) magnetic liposome. Scheme of liposome shape encapsulating iron oxide (right image). (d) Size distribution of ~140 nm diameter magnetic liposome, inset: photograph of magnetic liposome suspension in phosphate buffer (50 mM, pH 7.4).

Scheme for L-NIR-Fe_3_O_4_/OX assembly was shown in Fig. 1a with oxaliplatin as magnetic field responsive drug carrier for treatment of colorectal cancer. Uniformal ironoxide nanocubes of ~20 nm were prepared by thermal decomposition method (Fig. 1b) and oxaliplatin along with Cy5.5 were encapsulated in sterically stabilized PEGylated stealth liposomes. Liposomes were found to have a average diameter of ~140 nm ranging between 70-700nm (Fig. 1c and d) using TEM. Drug loaded magnetic liposome presented an oxaliplatin loading contents of ~2 % as defined by the following equation:


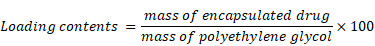


### Characterization of L-NIR- Fe_3_O_4_/OX

**Figure 2:**
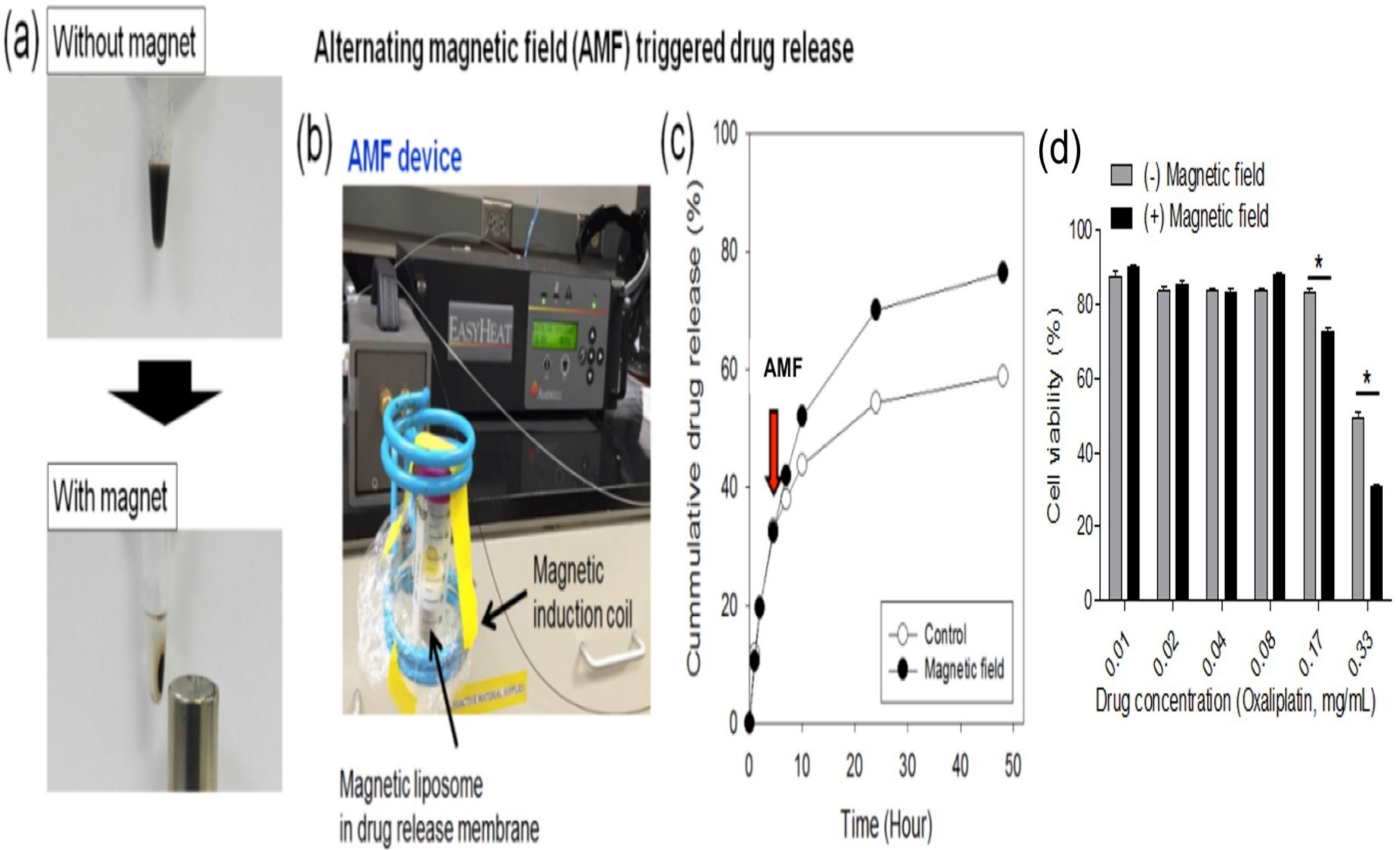
(a) Photographs of the magnetic separation of magnetic liposome, (b) Photograph of alternating magnetic field (AMF) induction device, (c) Cumulative drug (oxaliplatin) release from magnetic liposome when exposed (or not) to a high frequency alternating magnetic field (indicated by the arrow, at 5 h) at 37 °C in phosphate buffer (50 mM, pH7.4). (d) Viability assay on CC-531 cells with or without external triggering.

Magnetic liposomes were separated from solution within 30 minutes of attachment to a permanent magnet (Fig. 2a). To assess the influence of exposure of magnetic liposome to AMF, an oxaliplatin release study was performed (expressed as cumulative drug release, %) by using AMF device (Fig. 2b) and the released drug was quantified by using HPLC. An increase in drug release (~18%) was observed by HPLC method when the samples were exposed to the AMF compared to the untreated liposomes (Fig. 2C). Viability assay demonstrated that under high-dose oxaliplatin conditions (0.17-0.33 mg/mL), the cell viability after L-NIR-Fe_3_O_4_/OX treatment and exposure to AMF were significantly lower than those not exposed to AMF (*p*< 0.001) (Fig. 2D).

### Optical Imaging

**Figure 3:**
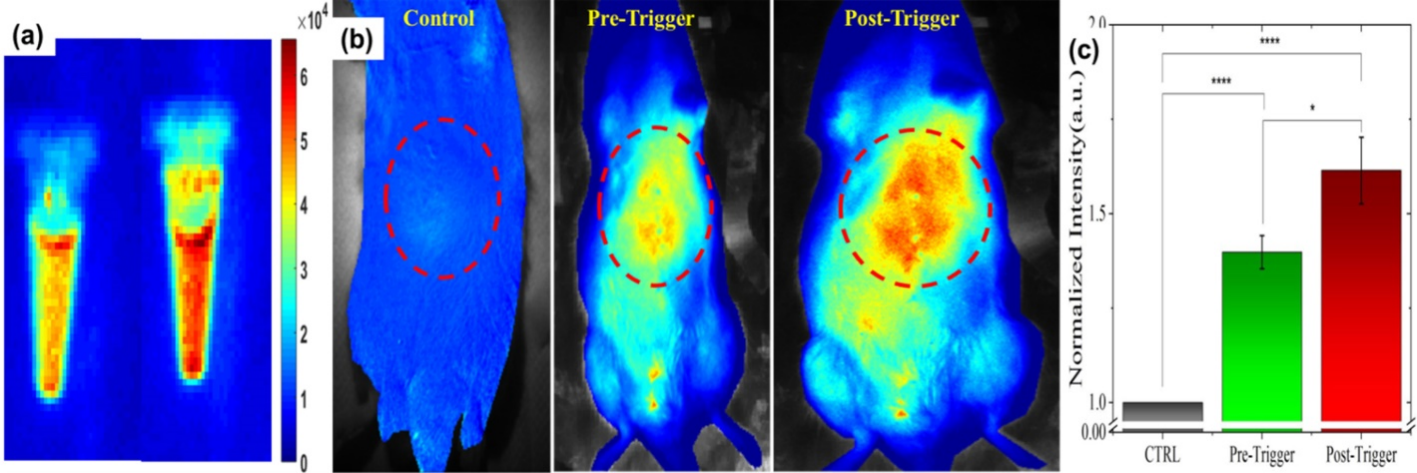
(a) Cy5.5 embedded in L-NIR-Fe_3_O_4_/OX imaged at pre and post AMF induction triggering. (b) Typical fluorescence image of rat captured at pre Cy5.5 infusion (CTRL), pre-triggering after Cy5.5 infused and post triggering. (c) Average intensity from ROI (red dotted circles in B) exhibits significant enhancement in fluorescence signal due to release in Cy5.5 post-triggering. (n=5; each group).

Fluorescent (Cy5.5) dye encapsulated in L-NIR-Fe_3_O_4_/OX offer a potential to image the cargo delivery in targeted region. Figure 3 depicts the fluorescence signal of nanoparticles encapsulated with 1 mg/ml of Cy5.5 captured before and after AMF induction triggering. Significant increase in fluorescence signal from post triggering indicates the contribution from released Cy5.5 (Fig. 3a). Average fluorescence intensity exhibited 11 - 15 % after the AMF triggering compared to untreated samples (Fig. 3a). To detect the *in vivo* impact of AMF triggered therapy with optical imaging, rats were imaged at pre-Cy5.5 infusion (CTRL), post Cy5.5 infusion (pre AMF triggering) and post AMF triggering as demonstrated in Fig. 3b. The enhancement in Cy5.5 fluorescence signal from post AMF triggering image clearly indicate the AMF induction effect in disrupting the Cy5.5 encapsulated nanoparticles via hyperthermia, and the subsequent dye release. For quantitative analysis, pre-Cy5.5 infusion image intensity (averaged from ROI) was used to normalize the pre-post trigger images (Fig. 3c). An average intensity plot depicted in Fig. 3c, demonstrates ~11% (ratio of post/pre -triggering) increase in post-triggered fluorescence signal than in pre-triggered.

### MR Imaging

**Figure 4:**
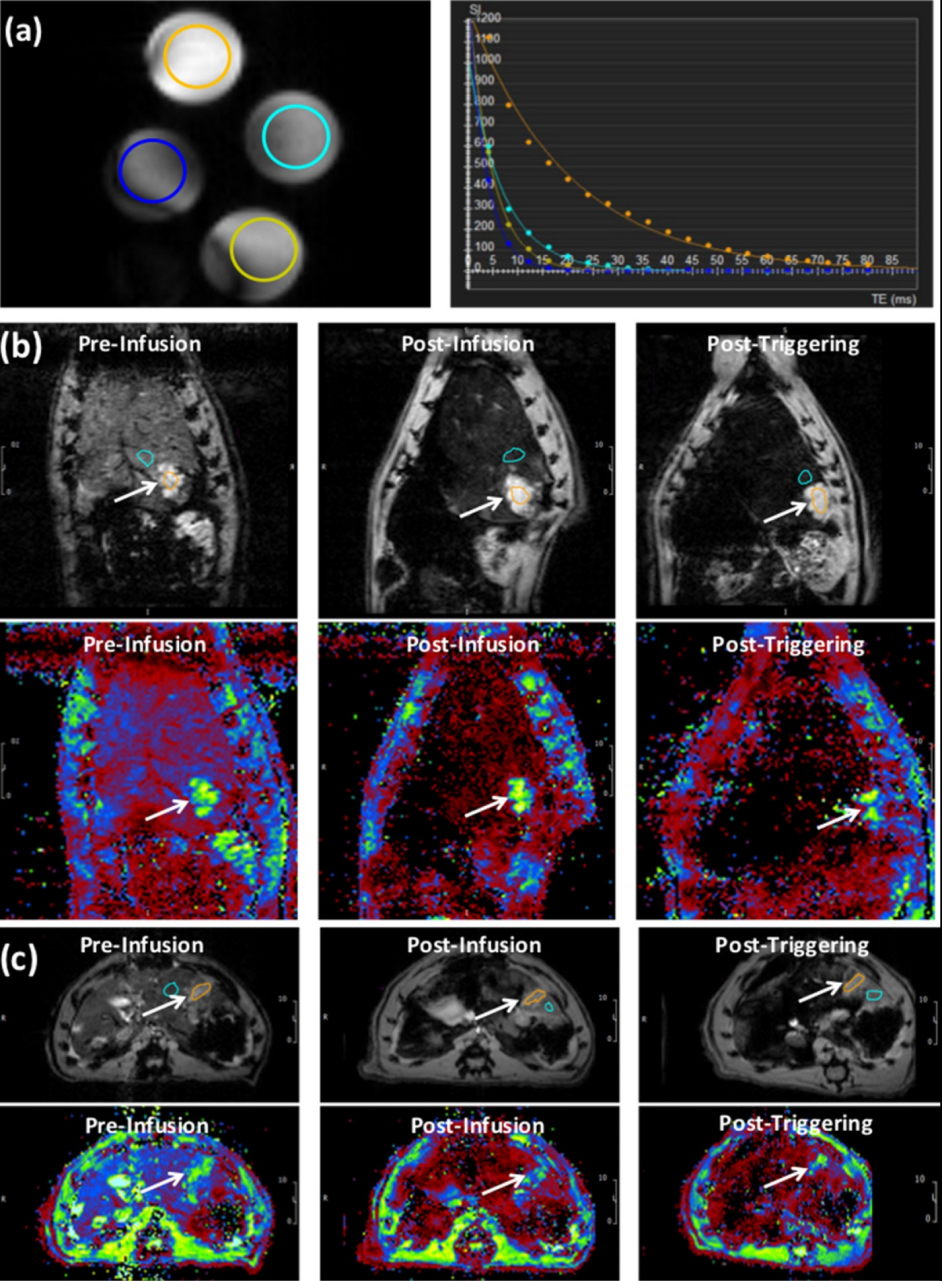
(a) T2* images showing cross-sections of tubes filled with different concentrations L-NIR-Fe3O4/OX doped in agarose gel. The circles color reflects decreasing signal intensity with increasing iron oxide nanoparticles (IONP) concentration, as shown on the right by the exponential fitting of the T2* versus IONP concentration. (b) Images (top) and T2* maps (bottom) of a coronal slice in a rat showing signal loss with liposome delivery (Fe) deposition in tumor region (arrows; post-infusion vs pre-infusion) and a further loss in signal with AMF post-triggering. (c) Similar results as in (b) but from an axial slice from a different rat. T2* color map (in ms) is shown in the bottom. Orange circle reflects tumor region and blue circles represent the respective regions using which normalization was performed.

The phantom T2* map showed decreased signal intensity with increasing Fe_3_O_4_ nanoparticles (IONP) concentration in L-NIR-Fe_3_O_4_/OX (Fig. 4a). T2* measurements showed decaying exponential relationship with IONP concentration, while R2* measurements showed linear relationship with IONP concentration (*R*^2^ = 0.924) (Fig. 4b). T2* values in the tumors (13.8±0.8 ms) were significantly (*p*=0.0004) different from those in normal liver tissues (4.7±0.7 ms) (Fig. 4c). The tumors T2* values significantly (*p*=0.003) decreased post L-NIR-Fe_3_O_4_/OX infusion to 12±0.6 ms. T2* further decreased after AMF triggering to 11.3±0.8 ms, which was not significantly different from the values before AMF triggering we saw a decreasing trend (*p*=0.2).

### Biodistribution

**Figure 5:**
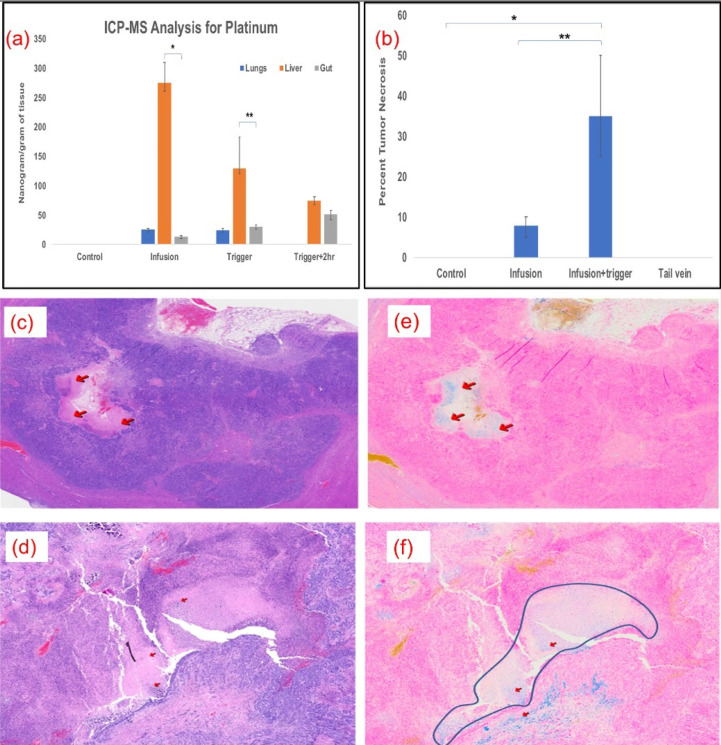
(a) Significantly higher levels of oxaliplatin was delivered to the liver and remained there compared to the lungs (*p*<0.001) and intestines (*p*<0.001); n=5 in each group. (b) Plot shows significant necrotic zones in infusion+ Trigger group (p<0.01). Arrows indicate the necrotic region in H&E staining (c & d) and Fe deposition with Prussian blue staining (e & f) (200X). Outline demonstrates necrotic regions in the field in infused and triggered animals, which serves as the surrogate for treatment efficacy.

Kinetics of oxaliplatin distribution was performed by ICP-MS analysis for platinum at various time points and in different tissues in MV and MV+Trigger group of animals. Platinum quantification served as our assay for oxaliplatin drug concentration. The liver platinum levels were high in the liver, as compared to lungs and gut indicating no off target delivery or systemic delivery immediately after infusion prior to AMF triggering (Fig. 5a). After triggering, the platinum levels decreased to 130 ng/gm of tissue in the liver suggesting release of the platinum and oxaliplatin from L-NIR- Fe_3_O_4_/OX (Fig. 5a). Furthermore, 2 hours after triggering, platinum levels in the liver declined to 75ng/gm tissue (Fig. 5a). The biodistribution demonstrates that higher liver uptake of platinum compared to lungs and gut.

### Histology

More than 50% tumor necrosis was observed in the MV and triggered group compared to untriggered or control groups (Fig. 5b). Prominent necrotic zones were observed in the implanted tumors of rats which received oxaliplatin via MV followed by triggering compared to those that did not undergo triggering and those which received intravenous oxaliplatin (Fig. 5c & 5d). Neither the control nor IV groups showed necrosis in the implanted tumors. Prussian blue staining revealed Fe deposition in necrotic zones and other tumor areas in serial histological sections, demonstrating that the nanoparticle deposited their cargo in the tumors (Fig. 5e & 5f).

### Survival

**Figure 6:**
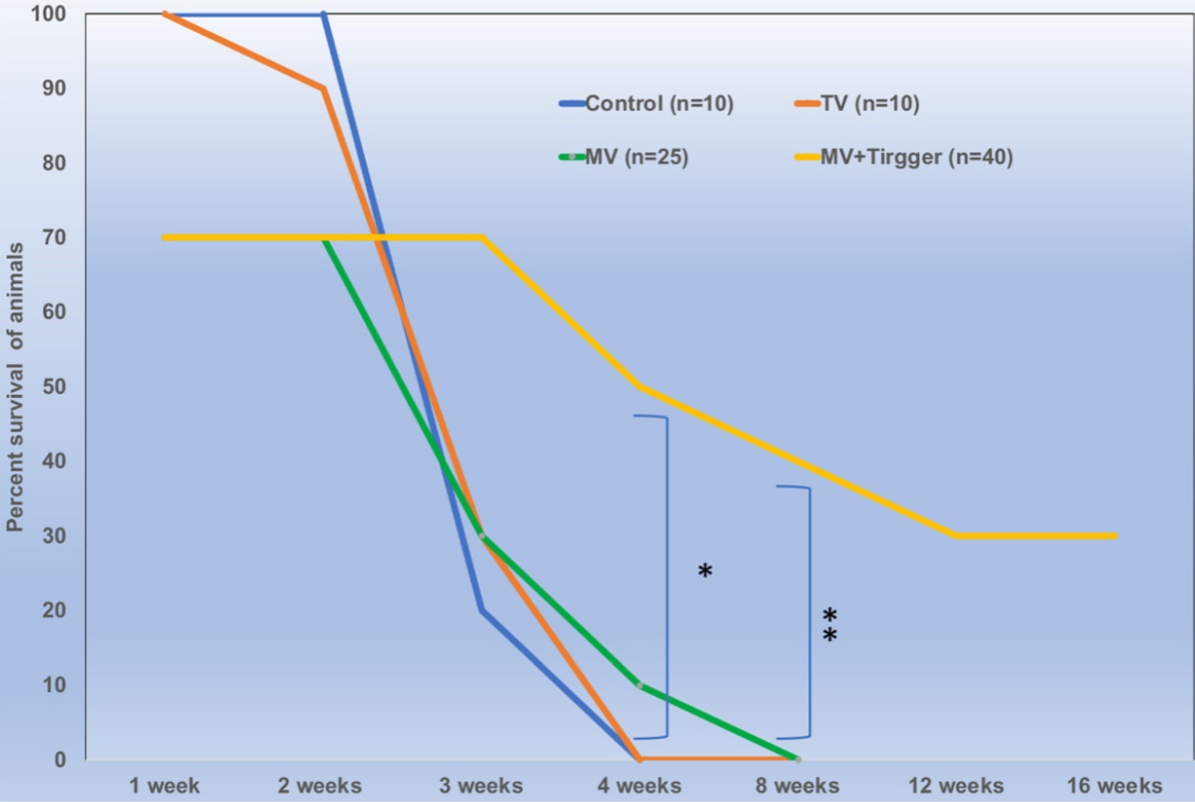
Locally delivered high concentration of oxaliplatin improves survival outcomes significantly (*p*<0.01) in colorectal liver metastasis tumor bearing rats.

There was no significant difference between the animals treated with single or multiple cycles of magnetic field triggers and the animals tolerated 3 trigger cycles with 100% survival rates. We have included 18 rats in MV group and 28 rats in MV+Trigger group for this study. The percent survival of animals treated with MV route delivery followed by triggering was significantly higher compared to untriggered and (IV) tail vein delivery (*p*<0.05) (Fig. 6). MV infusion related mortality is approximately 30% (Fig. 6) and this includes mortality during catheterization and immediate post-infusion. Nonetheless, we have noticed high mortalities in the initial experiments and later on we were able to achieve greater survival rates with ~10% mortalities. More than 30% of the animals in MV+Trigger group survived for 12 weeks (Fig. 6).

## Discussion

Several liposome preparations are now used as frontline treatment for a number of cancers, but only a handful of these have shown promising results in clinical trials 30-32. The loss of compound/drug activity in the body may be associated with heterogeneity of blood flow within the tumor, inhibiting its delivery to the tumor region and deactivation mechanisms irreversibly altering the chemistry of the compounds delivered 33. Several studies have demonstrated that water-soluble magnetic nanoparticle cores and anti-cancer drugs have limited encapsulation efficiency. In order to overcome this limitation, nanoparticles with magnetic core were attached to the membrane and cytotoxic drug was encapsulated in the core of liposomes 34. However, the challenge lies in destabilization and off-site release of drug when the particles are in the circulation for a long period of time. L-NIR-Fe_3_O_4_/OX is composed of a hybrid Fe_3_O_4_ core cluster, conjugated to a temperature sensitive liposome and oxaliplatin. Susceptibility difference between nanoparticles and surrounding tissues leads to local magnetic field gradients with proportional signal losses via R2 and R2* relaxation mechanisms. R2 and R2* measurements permitted *in vivo* quantification and visualize biodistribution of L-NIR- Fe_3_O_4_/OX concentration.

The ability is to track the release of cargo from liposomes aid in the confirmation of local delivery in the desired regions. Current fluorescence imaging method further adds information on tracking the liposomal cargo in the AMF applied region immediately after the trigger. Hyperthermia caused disruption of Cy5.5 encapsulated liposomes and the subsequent dye release results in an enhancement of Cy5.5 fluorescence signal. This helps in understanding the impact area and efficacy of therapy. In order to achieve a persistent killing of cancer cells, we have treated cells in “cycles” and demonstrated that L-NIR-Fe_3_O_4_/OX can be magnetically triggered at varying time points. Magnetic nanoparticles were known to be potential carriers for specific drug targeting to transport drugs to tumor sites. Interestingly, the release of oxaliplatin from L-NIR-Fe_3_O_4_/OX under the influence of AMF in 30 minutes was comparable to earlier studies on doxorubicin (DOX) release 35. This form of magnetically controlled oxaliplatin release has advantages over other conventional nanocarrier methods used for drug delivery such as diffusion, thermal response, etc. 36. The efficacy of magnetic fields for drug release in our study is indicated by effective intracellular uptake enhanced cell (CC-531) death within an hour of incubation in the presence of a magnetic field. Ferrite nanoparticle carrier conjugated with DOX significantly enhanced the release rate under magnetic fields by creating mechanical deformation, which generated compressive and tensile stresses to eject drug molecules. Notably, we observed a decrease in cell viability when measured by trypan blue after 24 hrs incubation. Other investigators have found similar results but shown the slow release of oxaliplatin from emulsion after 24 hrs of infusion and triggering liposomes 37. Very high levels of platinum detected in livers compared to lungs of the animals euthanized immediately after infusion provide evidence for the targeted delivery of liposomes. While locally infused and triggered release of oxaliplatin significantly improved the survival of CRLM tumor bearing animals, systemic adminstration of oxaliplatin did not show any effect. Survival benefit was also not seen in the MV infused animals that were not subjected to AMF. Similar studies on breast tumors with magnetic field 'on and off' system influenced dramatic release of camptothecin and DOX from nanoparticles and effective in reducing in tumor growth 38. Although 1, 2 or 3 cycles of triggering after L-NIR-Fe_3_O_4_/OX infusion did not show any difference in the survival benefit, the animals tolerated multiple triggers providing evidence to the safety of the procedure. The aim of localized delivery of chemotherapy is to increase local exposure to the drug, thereby increasing the antitumoral efficacy, while reducing exposure and toxicity in the rest of the body. Although intratumoral injection alleviates the off-target effects totally, the dosage that can be injected is severely limited. On the other hand, intraarterial or portal vein mediated administration of chemotherapy will significantly increase drug dose to the tumors in the liver. However, the drugs eventually enter into general circulation and exert off-target effects. Therefore, an external trigger used to release the drug only in the liver region prevents it from showing off target effects and increase the drug availability to tumor regions.

The current study had many limitations. Although we were able to image, quantify and note the benefits of triggered release of oxaliplatin, we have noted higher degree of infusion procedure related mortality. Additionally, IV L-NIR-Fe3O4/OX was not used as a control group because it was shown in the earlier studies that the circulating phagocytic cells along with cells belonging to the reticuloendothelial system ingest the liposomes and disrupt cargo release 39, 40.

In conclusion, external stimulus mediated locally delivered oxaliplatin improves survival outcomes in colorectal liver metastasis tumor bearing rats.
